# The potential use of digital technologies to enhance opioid agonist treatment in rural and remote communities of British Columbia, Canada

**DOI:** 10.1186/s12954-025-01302-z

**Published:** 2025-10-08

**Authors:** Amreetha Jayathilake, Kate Hodgson, Manal Mansoor, Kim Markel, Geoff Bardwell

**Affiliations:** 1 School of Public Health Sciences, University of Waterloo, Waterloo, ON Canada; 2 Department of Nurse Practitioners, Vancouver Coastal Health, Vancouver, BC Canada; 3 Harm Reduction Nurses Association, University of Victoria, Victoria, BC Canada; 4 British Columbia Centre On Substance Use, Vancouver, BC Canada; 5 Lift Community Services, Powell River, BC Canada; 6 Department of Medicine, University of British Columbia, Vancouver, BC Canada

**Keywords:** Opioid agonist treatment, Opioid use disorder, Overdose, Virtual technology, Autonomy, Surveillance, Rural and remote communities, Qualitative research, British Columbia, Canada

## Abstract

**Background:**

People who use drugs (PWUD) and Indigenous-PWUD in rural and remote communities are met with considerable barriers in access to opioid agonist treatments (OAT) in comparison to their urban counterparts. OAT is met with low rates of uptake and retention owing to clinic policies limiting access to take-home doses. Digital technologies may improve access to OAT for PWUD in rural and remote communities. The objective of this study was to understand willingness to use an asynchronous witnessed-dosing phone application among rural and remote PWUD.

**Methods:**

Qualitative semi-structured interviews were conducted with PWUD in rural and small communities in British Colombia, Canada (n = 32). Participants had to be ≥ 19 years old and have been on a prescribed OAT program within the 1-year of when the study began. A content analysis was completed on interview transcripts based on categories identified by the research team.

**Results:**

Participants described access barriers to OAT medications, which was contingent upon housing-related factors. Regardless of living in town, in coastal communities, or on Tla’amin Nation treaty lands, participants described accessing OAT medications as restricting, impractical, and stigmatizing. When presented with a potential alternative delivery method via an asynchronous virtual application, participants described potential improvements in not only access to their OAT medications, but overall quality of life through, independence and increased opportunities to engage in day-to-day activities. Participants noted potential difficulties in keeping mobile devices.

**Conclusions:**

Our findings help illustrate how current OAT prescribing practices and the challenges with requiring in-person witnessed doses for people living in rural and remote communities, exercising control over OAT patients, ultimately limiting the autonomy of PWUD. There is a clear need to implement virtual OAT programs to improve access to OAT medications.

## Background

The opioid overdose crisis continues to devastate Canadians, driven by fentanyl and its related analogs [1], benzodiazepines [2], and xylazine analgesics in the unregulated drug supply. In Canada, the province of British Columbia (BC), has the highest rates of illicit drug-related overdoses in the country, with rural and remote communities being disproportionately affected [3, 4]. Many people who use drugs (PWUD) are diagnosed with opioid use disorder (OUD). OUD continues to be treated with opioid agonist treatment (OAT) as the first-line treatment, which primarily includes methadone or buprenorphine/naloxone [1, 5]; however, in some Canadian settings it also can include slow release oral morphine [6], transdermal fentanyl [7], and both tablet and liquid injectable OAT (TiOAT/iOAT; i.e., hydromorphone and diacetylmorphine) [8]. Typically, prescribed OAT provisions mandate the observation of dose intake by a medical professional, usually a pharmacist, one to multiple times per day [5]. Although, OAT retention has shown to be associated with reductions in the risk of mortality for people with OUD [8, 9] and is the gold-standard of treatment for OUD, retention rates remain low [10]. Reasons for low retention rates for OAT in rural and small communities are owing to a multitude of intrinsic and extrinsic factors [1], stemming from prohibitionary laws on drugs [11]. These circumstances have propagated bio-power, a Foucauldian concept, that describes the ways in which governmental institutions have exercised control over the agency of PWUD, using several techniques [12]. Some such examples include, failure of medication dose to meet the patients’ needs, lack of technology for telehealth, travel restrictions, limited number of pharmacies offering OAT, decreased clinic and pharmacy hours, added out-of-pocket expenses, and stigmas associated with accessing OAT [1, 10, 11, 13]. Accordingly, policymakers and medical infrastructure, in BC, and Canada at large, need to advocate for more accessible methods of OAT to people in rural and remote communities [1, 14]. The use of digital technologies, is one such way towards improving access to OAT, by permitting flexibility in service provision [15].

Emerging digital technologies that intend to address harms associated with unregulated opioid use show promise in allowing an alternative to daily witnessed dosing. For example, in Vancouver, BC, a pilot project called The MySafe Program, utilized a highly secure biometric opioid dispensing machine placed throughout the city (in supportive housing and overdose prevention sites) [16], which allowed individuals to access prescribed safer supply (i.e., tablet hydromorphone) without having to visit a pharmacy or clinic [16, 17]. PWUD that accessed the MySafe machine experienced increased comfort when accessing prescribed opioids and allowed for greater autonomy and choice, namely through take-home doses and, in some cases, 24-h access [16]. Other research has explored the use of technologies during the COVID-19 pandemic to address existing restrictions to in-person clinical visits. For example, a pilot project in Washington State allowed video-observed therapy (VOT) via smartphones, when clients were accessing prescribed methadone [18]. Overall, clients experienced increased autonomy in their treatment regimen, through maintaining privacy with take-home supplies and increased their ability to maintain employment and other life priorities, during the months where VOT was allowed [18]. Moreover, VOT has also shown to be successful in other prescribed treatments where observed doses are also required, such as tuberculosis (TB) treatment, for adherence monitoring [19, 20]. For example, in the Canadian province of Alberta, VOT became standard practice for TB treatment during the COVID-19 pandemic. Patients experienced high satisfaction rates when using VOT, namely due to the elimination of adhering to restrictive clinic hours [20]. However, VOT use was restricted for use exclusively during COVID-19 pandemic restrictions on in-person care [20]. Although witnessed dosing for OAT prescribing is used to monitor medication diversion and overdose, rather than solely adherence, these examples showcase the value of use for digital technology in expanding access to essential medications and for the daily management of chronic illnesses such as OUD.

Recently, Sonara Health developed an asynchronous take-home web-application accessible on a mobile device for OAT. This application enables a person on OAT to access take-home OAT supplies whilst clinicians and pharmacists asynchronously observe appropriate medication use. Take-home medication bottles are fitted with tamper-evident labels to monitor dose-intake and address clinician and pharmacist concerns of mishandled and/or diverted medications. If a bottle has been opened or tampered with, the label (a QR code) breaks and patients are unable to scan it prior to taking their dose. The patient first opens the application and scans the QR label on the bottle and video-records themselves taking their dose. This video is then automatically uploaded to a secure repository for access by the patient’s health care team [21, 22]. The unique asynchronous feature means that the person on OAT does not need to have doses monitored in real-time. The novelty of this asynchronous feature for patients in rural and remote communities means that some of the known barriers of OAT access in these areas, such as transportation or limited pharmacy and clinic hours, may be addressed. Initial assessments on the usability and feasibility ratings of the Sonara application (app), describe positive experiences and improved quality of life [21]. Currently, the Sonara app is available in some cities in the United States, but to our knowledge, the acceptability of web-based applications has not yet been explored for people on OAT in Canada. Additionally, there are no known examples of the use of digital technology for OAT dosing in rural and remote communities. Accordingly, this study sought to examine the barriers in OAT access to PWUD in the rural communities of qathet, BC, and understand initial perspectives on the use of technology for OAT in addressing those barriers.

## Methods

This study is part of a larger 3-year longitudinal qualitative and ethnographic study in the qathet region that focuses on experiences of access and barriers to OAT for PWUD. This paper draws specifically from data from the follow-up interviews in year 2 of 3, which have a focus on the potential use of virtual applications for OAT. The qathet region is on the northern Sunshine Coast of BC and covers 5000km^2^ of land and is inclusive of various mainland communities as well as coastal islands with varied levels of accessibility by either ferry, water taxi, or private charters. The region is the traditional and unceded territories of Tla’amin Nation.^7^ In collaboration with community partners via the qathet Community Action Team, a rapid ethnography was employed. This methodology was utilized to obtain and deliver findings in shorter amounts of time [23], especially in considering the perspectives of using technology for access to OAT by people in the qathet region and deliver findings to the community more quickly. The community partners were consulted throughout the study, including when forming the objectives, creating the interview questions, developing the recruitment strategy, supporting recruitment efforts, and interpreting the data. The latter allowed the research team to write more authoritatively about the study population, notwithstanding the use of a rapid ethnographic design. This study was approved through the University of British Columbia/Providence Health Care Research Ethics Board.

Interview guides were co-developed with community advisory boards comprised of both Indigenous and non-Indigenous PWUD and/or those with experience on OAT in the qathet region. Given the longitudinal nature of this study, our follow-up interview guide used many of the same questions to examine changes across time (which we explore elsewhere). Prior to follow-up interviews, the study Principal Investigator and a clinical practitioner on our research team learned about the Sonara app at a conference and saw this as potentially addressing OAT access issues in rural and remote communities. Consequently, information about the Sonara app was shared with community partners at a meeting. Given the high interest in this app, it was decided to include questions about it in the follow-up interview guide to examine participant perspectives, willingness, as well as potential positive and negative implications of using an app to support OAT dosing across the region. Interviews ranged from 25 min to 1 h in length.

For follow-up interviews, participants were required to have completed a qualitative interview in year 1 of the study. Eligibility to participate in the study at year 1 required individuals to be ≥ 19 years old and have been on a prescribed OAT program within one year of the date of the interview. In collaboration with members of the Community Action Team and direct service staff (inclusive of individuals with lived/living experience), participants were contacted and invited to attend drop-in follow-up interviews. These were completed in February 2024. Given the close ties in the community, there were minimal challenges in contacting or connecting with study participants. In fact, we interviewed 32 of 33 participants and we were informed that the only participant who was lost to follow-up had passed away.

Members of the research team conducted the interviews at several different sites throughout the region in private rooms (e.g., private spaces in supportive housing, clinical offices). Participants provided written consent and were provided with $100 cash ($40 honoraria from the research team, $60 travel stipend from the qathet Community Action Team). Interviews were audio-recorded and transcribed by professional transcriptionists familiar with the subject area. NVivo 14 was used to organize and manage the interview transcripts. Each participant was assigned a unique identifier and interview data was deidentified. Data were stored on a password-protected and encrypted server at the University of British Columbia, only accessible by G.B., A.J., and M.M. Using NVivo 14, one research team member (A.J.) completed coding, with supervision (G.B.). A content analysis was utilized to determine patterns in the data [24]. Dominant categories were identified based on the frequency of common ideas reported in the data [24]. An inductive content analysis was used, in which coded categories were derived directly from the data as identified by the research team [24]. The dominant categories included the participants overall drug use over the past year, participants OAT regimen at the time of the interview, the impacts of OAT in their lives over the past year, changes to their OAT use within the last year, and categories specific to the Sonara app, including potential ease of use, security and privacy concerns, barriers in accessing the application, and overall willingness to enroll in such technologies. The research team’s positionalities as scholars and professionals in substance use, experience working with and studying substance use and related interventions, ultimately affected the completed analysis [25]. However, to improve researcher reflexivity, the analysis was completed collaboratively, allowing for feedback and conversations about interpretations from our community partners [26].

## Results

Of the total 32 participants, 24 participants were currently using prescribed OAT medications (i.e., hydromorphone, slow-release oral morphine, methadone, buprenorphine, and/or transdermal fentanyl) and eight participants were not currently enrolled in any OAT programs. Twenty-one had housing, either supportive housing or private residence, whereas eleven were experiencing homelessness. A complete description of participant socio-demographic characteristics is referenced in Table 1. Participant perceptions on OAT access over the year and initial thoughts on the Sonara app are described below.

**Table 1:** Participant socio-demographics (n = 32)

Age	
Mean	45.9
Range	22–63
Gender	
Woman	10
Man	20
Undisclosed	2
Race/ethnicity	
Indigenous	5
White	26
Undisclosed	2
Housing	
Supportive housing	11
Apartment	2
House	3
Shelter	9
Other	8
Preferred drug	
Crack cocaine	6
Crystal methamphetamine	7
Heroin	2
Fentanyl	23
Other opioids	1
Benzodiazepines	3
Cannabis	1
Other	7
Currently prescribed OAT	
Yes	24
No	8
Prescribed OAT	
Methadone	8
Kadian	7
Dilaudid	15
Sublocade	2
Fentanyl patch	8
Overdose in past year	
Yes	13
No	15
Undisclosed	4

### Current OAT access experience

Most of the participants lived in Powell River (i.e., the largest community in the qathet region of ~ 14,000 people). A key finding was that OAT access varied according to housing precarity. For participants living outside of supportive housing, OAT regimens included daily pharmacy and/or OAT clinic visits at minimum once per day, although for many, multiple daily visits was a reality. Accessibility barriers were especially heightened for those that lived in the surrounding remote areas that lacked OAT clinics or pharmacies, forcing participants prescribed OAT to travel long distances daily which significantly impacted adherence. For example:

“…*the ferry rides are just too draining. Like I live 22 km away from the ferry already, so getting to the ferry is hard enough, let alone having to do a four-hour trip once you get there, and then another hour home, like the whole day turns out to be an eight-hour trip.”* (P18)

For participants living within supportive housing, OAT medications were most often delivered by local pharmacists at a specific time during the day. Although participants described OAT delivery as beneficial, these predetermined OAT schedules were not without critique. For instance, one individual described the delivery schedule as “*goofy*” stating “*like one o’clock in the afternoon and six in the evening… I’d like my first dose to be in the morning*…” (P6). Meanwhile, others required their OAT dose at night, but “*…pharmacies close… well, what do you do from eleven to seven?*” (P23). Additionally, as the pharmacist’s delivery schedule is not individualized, some participants described not receiving their OAT medications as prescribed. For example:

“*They don’t have times, like they don’t have it in the morning and then noon and then night, they just do it when…twice a day, right. That’s it. They are both two hours apart or something; what good is that, right? And you’ve got to do it or else they don’t give it to you. Yeah, it’s stupid, real stupid. It’s going to take somebody to OD or something like that before they stop doing that stupid practice…You’re supposed to take them…eight hours apart.*” (P5)

To add to this unpredictable schedule, in cases where the individual is not within the facility when the pharmacist arrived at the housing facility, the individual would miss their OAT dose for the day. For example, as participants describe “*it gives [us] no time to do anything else or else [we] don’t get [our] meds,*” (P5) or that it is “*hindering…in life, like stopping [me] from getting a place and stopping [me] from being able to work…because I would be here so many times a day*” (P4). Additionally, amid conducting these interviews at supportive housing, interviews had to be paused so interviewees could get their OAT dose before the pharmacist left. If we had scheduled these interviews at another location as we did for many other interviewees, these participants would have missed their OAT dose. This speaks to the limiting nature of OAT schedules and the individuals need to be available at any time of day for delivery interrupts the opportunity to engage in other health or socially productive activities.

Additionally, OAT guidelines for prescribers in the province of British Columbia permit access to take-home doses (i.e., carries). However, in this study of the 32 participants, only two had access to take-home doses (i.e., carries). Most participants were unsure of the reason other than the prescriber’s preference and possibly conflating previous experiences onto other patients. For example, when asked about getting carries participants stated: “[Prescriber] doesn’t give anyone carries. I don’t know why” (P8), “[No], I don’t think anybody does these days” (P28), and “[prescriber] just doesn’t—just because that’s [their] personal experience with [their] other clients” (P6). The one participant that was prescribed carries stated “finally, since I’ve been on them for five years, it’s finally went that way” (P32). This overall difficulty in accessing OAT led many participants to conclude that their adherence to an OAT program as unnecessarily difficult, owing to restrictive prescribing practices. We observed that many participants had discontinued OAT over the past year, as one participant described “*it just seems easier to use street drugs, even though it’s dangerous*” (P11). The limitations on take-home doses that these participants have experienced perpetuates dependence on pharmacists and clinics and limits their autonomy by forcing individuals on prescribed OAT to focus on one thing only—accessing their OAT medication. As described by one individual, visiting or waiting for the pharmacist for prescribed OAT is akin to meeting one’s drug dealer to get one’s fix for the day:

*“…people are using the medicine and the drugs because there is nothing in the way that they’re attaining the medicine that’s changing the thing that needs to be changed to get off the drugs. It’s not just the drugs. It’s the whole structure of your day, it’s the structure of your life. It’s how everything, they say getting clean is easy, you just have to change everything. So, how is coming into town every day to get medicine or coming into to town to see your drug dealer every day, you’re doing the same fucking thing; it’s not going to work.”* (P20)

### Perceptions of using technology to access OAT

Given that daily witnessed dosing was often a barrier in accessing prescribed OAT, participants provided their perspectives on the potential use of a virtual option to access OAT (i.e., the Sonara app, described above). For most participants (n = 27), the Sonara app was perceived as a more convenient and accessible mode for OAT medication adherence, expressing how eliminating the need for travel to a pharmacy or OAT clinic would be the key determining factor in terms of medication access and adherence. For example, for those receiving tablet hydromorphone (oftentimes alongside other long-acting OAT):

*“I don’t get all my pills right now. In order to get all my pills throughout the day, I have to see the doctor—or see the pharmacist four times… So, if I had all my pills, of course it would definitely help me get all my pills throughout the day because now it’s all here. I don’t have to worry about delivery. I don’t have to worry about getting to a pharmacist. Don’t have to worry about being here when the pharmacist shows up. I mean it would absolutely help me get my pills throughout the day. Definitely improve it.”* (P6)

For some individuals, particularly those living out of town on Tla’amin treaty lands, which is approximately a twenty-minute drive to Powell River by car and has limited public transportation availability, access to prescribed OAT is further restricted as at the time of these interviews, Tla’amin Health Centre did not provide access to certain OAT, such as transdermal fentanyl and hydromorphone, owing to the additional resources required to have this service available. Those living in Tla’amin must travel to town to obtain their prescribed OAT. Due to this, some individuals continually missed a portion of their daily OAT dose. As one participant describes, “*yeah, I only take it once a day but…I was on three times a day… but I just never ended up taking it three times a day*” (P31). Accordingly, the Sonara app would provide “*less hassle*” and “*save [one] from coming into town, especially when it’s three times a day*” (P31).

To add to this, participants described how a virtual application for OAT may address experiences of daily stigma within their community around drug use and prescribed OAT. For example, one participant explained “*you’re not standing in a lineup asking for the drug in front of people. There is a lot of stigma around even OAT drugs, you know”* (P18). Currently, participants describe how they experience stigma when accessing their OAT medications stating “*yeah, there’s a lot of stigma down there… just a little bit judgemental people down there*” (P31). For one individual, the experience of stigma is pre-determined prior to even accessing the pharmacy:

*“just small-town ideology we’re walking into, just when walk to a clinic and then walking out I know people see you and people talk about it, people know that you’re there getting your medication. And there’s unfortunately it’s a, you know, people like to gossip about that kind of stuff.”* (P33)

Accordingly, the Sonara app may aid in reducing the stigma that these individuals experience on a day-to-day basis. Overall, participants had a positive response toward the potential use of the Sonara app, including perspectives on privacy. Almost all participants were not concerned with privacy as related to uploading a video of dose intake onto the app’s secure repository (n = 31). For example, one participant described how the benefit of limited in-person visits would outweigh concerns about privacy, stating “*I think the good part of being not tied down somewhere would overrun the part of the privacy issue*” (P19), knowing that these would be kept confidential and be accessible by only the individuals’ health care providers. However, one potential limitation that was consistently presented was the idea of being able to keep a phone for an extended period. Theft and the need to sell one’s belongings decreased participants’ confidence in being able to maintain their virtual OAT dosing long-term. For example, one participant mentioned “*I can’t keep a phone for a month. I’ve got to buy one every month because they steal it*” (P5). Another participant stated, “*I mean people around here they have a phone maybe a week if they’re lucky, and it gets sold, right*” (P6).

Overall, participant willingness within this sample to utilize the Sonara app for OAT uptake was high; at the end of one interview, a participant stated “*I think the app is an awesome idea. Please, please, please, please make sure it works, and just anything, anything you need to get it done*” (P20). This speaks to the potential impact that virtual dose-monitoring could make for people living in rural and remote communities.

### Gaining independence through using technology to access OAT

When describing their perspectives on the Sonara app, many individuals described the sense of independence that could be gained through access to their OAT using technology. For example:

“*That’s a big door, that is a big way to open the door for a working-class citizen of any sort, you know what I mean? I mean cause all those years that I made it work, I made it work, but it was always a barrier of some sort.*” (P32)

With current prescribing practices, some individuals described witnessed dosing as infantilizing. For example, one participant stated “*the pharmacist…treating us like little children, kind of thing. You had to open your mouth when you were doing Dilaudids and stuff like that*” (P23). Additionally, participants also describe how OAT regimens monopolize their daily routines, for example: *“I have to wait here to get my Dilaudid in the morning and then I’ve got to go there and pick up six more and then I’ve got to come back here and pick up six more*” (P5). Therefore, being given the opportunity to manage their own OAT medications from home and no longer being restrained by pharmacies or OAT clinics’ schedule would provide a sense of agency. For example: “*it gives a person more of a sense of empowerment that they can do their own medication and take it like it’s prescribed, you know, give them the power to do that, instead of taking it away from them…”* (P18), referring to standard prescribing practices of restarting OAT medications at a lower dose due to missed doses. The Sonara app would allow the freedom to work on their own schedule and reduce dependencies on pharmacies and clinics to obtain daily OAT medication. For example:

*“Awesome, yeah, I love that because I want to be more in control of what’s going on. I actually don’t even want to be doing the dope. So, that’s really good. Not only that but it frees you up to be able to go and do other things because here we’re going oh well, we’ve got to wait for meds in the morning and this and that. Then if we miss it, we’re going oh my god now we’ve got to go down there, you know what I mean.”* (P9)

This participant perspective showcases how providing an alternative form of dose-monitoring through technology can empower a sense of autonomy to PWUD. Additionally, participants described how the Sonara app could provide more realistic opportunities for employment or education. For example: “*I’m a [construction] worker by trade and I like doing it and I haven’t been able to do it because I can’t leave…So I find myself waiting on my medications and I’m not doing or accomplishing anything”* (P12), highlighting another gap that the Sonara app could fill.

## Discussion

Overall, participants showed a high interest and willingness to use the Sonara app in aiding their OAT access, illustrating how such technologies may not only help address some of the physical barriers in OAT access, but further propagate independence and opportunities beyond environments of drug use that could lead to a positive cascading effect on overall health and well-being by improving adherence to OAT. Participants described how using the Sonara app could reduce travel requirements for visiting a clinic or pharmacy in person, allow for more individualized dosing schedules, and reduce feelings of stigma associated with accessing OAT in rural and remote communities. While negative perspectives were limited, participants did identify challenges with consistent access to mobile phones, especially amongst those experiencing housing instability. This finding is consistent with Tsang and colleagues [27] who described how the acceptability of the use of technology by PWUD for overdose monitoring was limited by mobile phone availability, especially for PWUD experiencing insecure housing, increasing their vulnerability to theft [27]. Albeit this issue may be diverted by educating app users that Sonara app is web-based application, meaning that users can log-in on *any* device with internet connection and is not limited to a single device [22]. At this time, an app like Sonara Health may not be able to address the issues of mobile phone theft. However, if implemented, education on protection and safety of personal belongings may be well suited between the clinician and patient at initial set-up with the Sonara app. For instance, clinicians or health care staff may suggest storing their mobile device in a lockbox that also stores the individuals’ take-home doses. Nonetheless, these findings demonstrate largely positive perspectives on using mobile technology to aid in OAT access.

There is a body of research demonstrating how using technology for OAT access can improve individual autonomy [16, 18, 20, 28]. For example, participants from the MySafe project in Vancouver, utilized a biometric machine that scanned patients’ hands to dispense their prescribed opioids daily. Despite the embedded surveillance mechanisms (e.g., video recording, participant tracking), participants were not concerned with privacy in this regard, which the authors suggest is due to increased individual autonomy gained by the mode in which the prescribed opioids were accessed [16]. Our participants had similar perspectives on the potential use of this new technology, by describing how the Sonara app would help gain autonomy in their lives through their ability to take medications within the privacy of their own home and on their own schedules. Additionally, by using the Sonara app, participants described how they may have more opportunities for employment, freedom for travel, and other daily activities. It is documented elsewhere that methadone maintenance treatment has a negative impact on employment initiation [29], likely due to prescribing practices requiring individuals to attend a pharmacy or clinic daily to obtain observed doses. As such, providing new ways to allow witnessed dosing, through technology, would likely lead to greater engagement in employment, among other personal, social, and professional activities.

Despite current guidelines that permit increased accessibility to take-home doses, especially for those in rural and remote communities [30], for many prescribers, take-home doses bring concerns of increased risk of medication diversion, mishandled supplies, overdose, and difficulties in judging stability amongst their patients [12, 18, 23]. These examples could be some of the reasons that most participants in this study did not have carries. Providing an alternative access method, such as virtual-witnessing technologies like the Sonara app, may not only improve access and adherence to OAT but also aligns with prescriber concerns for patient safety due to the program design and its use of tamper-evident labels. Virtual technologies are a secure and confidential way to permit virtual witnessed dosing. Additionally, as described in this study, nearly all participants were not concerned about privacy with uploading videos to the Sonara app’s secure repository for view by their healthcare team. However, the continued requirement of witnessed doses bodes questions on how surveillance is ultimately embedded within the design of OAT practices [31]. As described by our participants, because the use of technology would allow for freedom to accomplish activities in other aspects of everyday life, outside of those related to drug use, the continued requirement of witnessed doses through video is justifiable.

Foucault’s theory of bio-power helps to understand this potential illusion of free will by PWUD. Foucault describes bio-power as “historically entrenched institutionalized forms of social control to discipline bodies” [12, 32]. In other words, it is the subjugation of one’s agency at both the individual and population level [12]. The continued practice of surveillance imposed by regulatory and evaluative policies [31], including federal regulations that restrict OAT prescribing to limited provider types [33], leads to stagnant prescribing patterns and perpetuation of clinical biases from one patient to another, as described in this study. Accordingly, virtual OAT services are masked by potential notions of freedom, through the ability to take OAT medications within the *privacy* of one’s own home, or the lack of hinderance caused by multiple pharmacy or clinic visits per day. As such, although the use of technology may provide many benefits to PWUD on OAT medications, we must question the necessity of the surveillance design embedded within such applications, as it may maintain a power imbalance between the person using the OAT medication and the health care provider. Namely, through observed dosing, health care providers maintain an ability to “mobilize PWUD towards desired outcomes of the prescriber or achieve greater compliance (e.g., withholding take-home doses, threatening involuntary treatment, discontinuation)” [31].

Moreover, for individuals who’s desire is to reduce or abstain from unregulated drug use, a common practice is to avoid “people, places, and things” that can trigger one’s desire to use their substance of choice [34]. As one participant expresses so powerfully within this study, the structure of accessing OAT does not allow individuals to change their habits and drug-seeking behaviours, rather forces them to maintain these behaviours. The need to visit a pharmacy or clinic in-person one to multiple times per day and see other PWUD also obtaining their OAT can trigger maintenance or relapse into unregulated drug use. Accordingly, virtual technology for daily medication intake enables the opportunity to separate oneself from familiar environments and allows individuals more autonomy and control over their daily routines and subsequent drug intake.

Finally, it is important to point out that one quarter of participants discontinued their OAT medications from baseline to follow-up. This further supports research that indicates how daily witnessed dosing is simply not feasible for OAT program adherence in rural and remote communities [33, 35], although discontinuation may also be affected by other factors including precarious housing. Current guidelines for the treatment of OUD regulate patients to take‐home doses only once the following conditions are met in unison: urinalysis free from unregulated substances, cognitive and emotional stability demonstrated through observations taken at OAT appointments, and no indication of injection drug use [33, 36]. Understanding the feasibility of technologies to improve access to OAT requires an understanding of systems levels policies and regulation on OAT prescribing, and further highlights the need for health care professionals to increase access to OAT and similar programs to reduce the burden of travel requirements, stigmatization, and service gaps for PWUD in these areas [37].

### Limitations

Although the study aimed to recruit a diverse study sample to represent the qathet region, their perspectives may not represent all the experiences of people on OAT across the qathet region, including people who may appreciate and benefit from daily clinical visits. As such, future research should aim to identify other perspectives related to OAT access and delivery in rural and remote regions. This would also help in identifying which subpopulations may or may not benefit from virtual OAT applications. This portion of the study also was exclusive to interviews with people with lived/living experience of OAT access and it would be integral to also capture the perspectives of pharmacists, nurses, physicians, and other healthcare staff across the qathet to understand their perspectives about the potential benefits and challenges with the use of the Sonara app to augment current OAT programs. As such, future research should be conducted with these groups, which will be integral to understanding feasibility, design, and any future implementation efforts. Lastly, it is possible that participants displayed social desirability bias, in which the participant may have provided favourable responses [38] to questions related to the Sonara app. Given that this was a willingness study, perceptions regarding potential versus actual use may skew participants to speak more favourably about the benefits of the application. As such, it would be important for future implementation research on the Sonara app to revisit these themes to confirm or modify the findings from this study.

## Conclusions

This study examined participant perspectives of the use of technology in accessing OAT. While not without its potential problems, there was an overall high willingness to use a virtual application for OAT dosing. Considering the imminent risk of overdose and overdose-related deaths in rural and remote communities in Canada, finding novel ways to improve access to OAT medications all while increasing individual autonomy is crucial. Although, the use of technology may pose benefits to PWUD by creating opportunities for improved access to OAT medications, we suggest paying caution to surveillance practices utilized in virtual applications.

## Data Availability

The datasets generated and/or analysed during the current study are not publicly available given the sensitive nature of the topic, as they contain confidential information that could compromise participant confidentiality and consent.

## Appendix 1

See Table 1

## Appendix 2: Year 2 follow-up interview guide

**Table:**

Section I: Changes to context	
Primary	Probing
Have there been any changes in your housing situation?	*How has it impacted your drug use and access to OAT? What has transportation been like in the area? Have you run into any issues? Who do you live with and does living with them have impacts on your drug use or OAT access?*
Have there been changes in your drug use since the last interview?	Describe your drug use. What does an average day look like to you? Tell me about what substance use services you are accessing lately How do you access harm reduction services and supplies?
Had you overdosed in the past year?	*Tell me about the circumstances that lead to your overdose* *Did anyone respond? What happened?* *Had you taken your OAT medication that day? why/ why not?*
Can you describe any changes in the drug scene over the last year?	*For example, costs, availability, modes of consumption, new substances?* *Can you tell me how these changes have affected you?* *What do you do to protect yourself from overdose and other harms from the street supply?*
Section II: Current OAT	
Are you currently enrolled in OAT right now? E.g., methadone, buprenorphine, Kadian, tablet or injectable hydromorphone, patch fentanyl, etc	*Why or why not?* *If not on OAT, what are some reasons why you are not taking OAT medications? (Probe around individual health, employment, family, other factors)* *If not on OAT, are you currently using any drugs or are abstaining from drugs?* *What led to this change? (probe around above topics) What support do you have (e.g., medical, psychological, social)?* *If yes on OAT, what are you currently taking and what is your daily dose?*
We learned from nursing staff that some people who were originally prescribed liquid injectable hydromorphone switched to tablet hydromorphone. Did this happen to you?	*Was this decision made by you or your physician?* *What was the rationale for this switch?* *Do you have a preference for tablets compared to liquid? If so, can you tell me the difference?* *(probe on physical feelings when injecting tablet vs liquid)*
Last year, you told us you were prescribed and taking [INSERT DRUG NAME HERE], has that changed and if so, can you tell me about what led to this change?	*(Access) What motivated you to seek other forms of OAT? Who prescribes it to you? How do you access the medication? Do you find it accessible? Do you think it’s sufficient for your needs? why/why not?* *(Discontinuation) Have you discontinued any OAT medications since our last interview? What motivated you to discontinue them? Was it your decision to discontinue them? Was there anything going in your life when you discontinued them? Any positive/negative impacts of discontinuing this OAT medication?* *(If on multiple OAT programs) How do your new OAT medications interact with your other OAT? Any negative side effects? Do you think the combination of both is what is helping you achieve your goals? How often do you use street drugs while on OAT? / Has OAT reduced your street drug use or had impacts on any other aspects of your life?*
Have there been any changes in how or where you received your OAT medications?	*For example, the iOAT clinic has moved to a new space. How does it compare to the other space? Tell me what you like about the new space and what you don’t like. (probe around built environment impacts)* *Do you receive delivery? Or where do you access your medications now? Tell me about what that looks like. Do you experience stigma or feel judged where you pick up OAT? If yes, can you tell me more about that? Is where you pick up OAT discrete enough for your comfort? If not, How would it be different to meet your needs?* *Does attending a clinic where other people also use drugs affect your drug use at all? If yes, what would help your situation?*
Do you get carries for any of your medications?	*Why/why not?* *How does this impact you? (e.g., employment, school, families, appointments, freedoms)*
Impacts: Have you noticed any other changes – either positively or negatively – over the last year related to your enrollment in OAT?	*Drug use?* *Employment/finances?* *Ø If yes, is your work schedule compatible with your OAT dosing schedule?* *Ø If no, is being a part of the OAT program a barrier to getting a part-time/full-time job? How?* *Engagement with other health and social services?* *Ø Have you found it easier to keep up with other appointments with your physician/social worker/housing provider/employment?* *Ø Have you accessed any other forms of health or social support since the last interview? Which ones?* *Encounters with police/criminal justice?* *Relationships/family?* *Health impacts?* *Ø Mental health impacts?* *Ø Social health impacts?* *Ø Physical health impacts?* *Ø Spiritual health impacts?* *How you spend your time?*
Section III: iOAT EXPERIENCES	
(If they have started the iOAT program after baseline) Can you please describe both the pros and cons of being in the iOAT program?	*What do you like about it? Have you noticed any improvements to your health and wellbeing since being enrolled? Have you noticed improvements in your social life since you’ve enrolled?* *What don’t you like about it? Have you noticed any negative side effects? Or any impacts on your health and wellbeing since being enrolled?*
How has iOAT impacted your substance use?	*Have you found you’re using more street drugs? Or less street drugs? If so, why? Has the way you use drugs changed? (ie. Have you started smoking instead of injecting?)*
Has it had an impact on any other area of your life?	*Probes: Work, family, social life, health service engagement, housing, *etc
How has iOAT changed the way you engage with health and social services?	*Is iOAT a space you use for primary care? Have any health issues emerged since starting iOAT? Have you had to go to the emergency room/hospital since starting iOAT? Are you going to Lift services more frequently because you’re on iOAT? If so, has that connected you with other social services available through Lift? If so, what services?*
SECTION V: CULTURAL SAFETY (Indigenous participants only)	
Have you received OAT or other health services from Tla’amin Health in the last year?	*[if YES] Can you tell me what your experiences have been like accessing care at Tla’amin Health Centre. What do you like about it/What do you not like about it?* *What prompted you to access health services or OAT at Tla’amin Health Centre? What is it like accessing OAT there? Is your prescriber now based there? Does your provider provide culturally appropriate care? Why/why not?* *Do your other providers know that you use substances? Why/why not? Would you feel comfortable disclosing your use to service providers? Why/why not?* *[for YES and NO] If there was a pharmacy located at or near Tla’amin Health Centre, would you access it for your OAT or other medications? Why/why not? What would be some benefits? What would be some challenges?* *Are there any other harm reduction services offered to you there? If so, what are they? If not, what is missing and would you want to access these at Tla’amin Health Centre?* *Can you tell me about what you like / don’t like about Tla’amin Health Centre? Is there anything you would change?*
Have you accessed health services anywhere else in the last year? For substance use or other health issues?	*If no, why not?* *If yes, where did you go?* *Can you tell me about any experiences of discrimination you faced when accessing any health services in the last year?* *Can you tell me about any situations where your Indigenous identity has created additional obstacles or delay in accessing care? What about in accessing substance use care specifically? (e.g., OAT, treatment, detox, overdose prevention site, *etc*.)*
*Are you involved in the ajimet harm reduction circle?*	*If no, why not?* *If yes, can you tell me how you became involved?* *(Probe around impacts on socialization, substance use, connections to healing, resources, *etc*.)* *How might ajimet be improved to meet your needs?* *Is there anything else you’d like to share about your involvement in the ajimet harm reduction circle?*
SECTION VI: NOVEL OAT SERVICE DELIVERY	
At our last round of interviews and focus groups, we learned that there are a number of challenges with attending a clinic or pharmacy every day for people to receive and take their OAT medications. At a community meeting, people expressed an interest to find a way to address the issue of attending a clinic every day to have someone witness you take your medication. A phone application called Sonara was presented at the community action team meeting as a potential solution	*I am now going to explain to you what this app is and then ask you a series of questions regarding its potential use by you and others across the qathet. If you want me to repeat anything including the description, please ask* *[show participant Sonara website on laptop to assist with explanation/visuals]* *The app is called Sonara. You would have a week’s supply (or more) of your methadone or other OAT medication in bottles at home. When it is time to take your dose, you would scan one of the bottles, remove the lid, and record a video on your phone of you taking your dose. When you are done, the app would upload the video to a server at your clinic or pharmacy so someone can review the video to see that you have taken your dose* *Is this something you would be interested in? why/why not?* *Do you think this app would address some of the challenges you mentioned earlier in the interview (probe based on response; e.g., interfering with work, school, leisure, family, social life, *etc*.). Why/why not?* *If this app were available in qathet, would you enroll in a program and opt out of your current clinic/pharmacy visits and/or medications delivery? Why / why not?* *How might it affect dosing or medication adherence?* *How might it affect your relationship with your doctor or other clinician (NP, RN)?* *What might be some challenges with it?* *Do you have a cell phone or internet access where you live or stay? Or is there internet access close by?* *Would you have any concerns about the security of your medications? For example, if someone might steal them or if a family member or loved one might take them. Why/why not?* *Would you have any concerns over privacy and confidentiality either from dosing at home or where you stay or from uploading videos to a secure server?* *If a clinic started offering this program in qathet, would you enroll? Why/why not?* *Do you have any other thoughts about the potential use of this application?*
Is there anything else you would like to share about your drug use, OAT programs, and/or Indigenous experience that we haven’t covered?	*Thank you*

## Abbreviations

PWUD: People who use drugs
B.C.: British Columbia
OAT: Opioid agonist treatment
OUD: Opioid use disorder
VOT: Video observed therapy
App: Application
